# QCM-Arrays for Sensing Terpenes in Fresh and Dried Herbs via Bio-Mimetic MIP Layers ^†^

**DOI:** 10.3390/s100706361

**Published:** 2010-06-28

**Authors:** Naseer Iqbal, Ghulam Mustafa, Abdul Rehman, Alexander Biedermann, Bita Najafi, Peter A. Lieberzeit, Franz L. Dickert

**Affiliations:** Department of Analytical Chemistry, University of Vienna, Waehringer Strasse 38, A-1090 Vienna, Austria; E-Mails: naseer.iqbal@univie.ac.at (N.I.); ghulam.mustafa@univie.ac.at (G.M.); abduhl.rehman@univie.ac.at (A.R.); alexander.biedermann@univie.ac.at (A.B.); bita.najafi@univie.ac.at (B.N.); peter.lieberzeit@univie.ac.at (P.A.L.)

**Keywords:** quartz crystal microbalance, chemical sensor, molecular imprinting, herbs, terpenes

## Abstract

A piezoelectric 10 MHz multichannel quartz crystal microbalance (MQCM), coated with six molecularly imprinted polystyrene artificial recognition membranes have been developed for selective quantification of terpenes emanated from fresh and dried Lamiaceae family species, *i.e.*, rosemary (*Rosmarinus Officinalis L.*), basil (*Ocimum Basilicum*) and sage (*Salvia Officinalis*). Optimal e-nose parameters, such as layer heights (1–6 KHz), sensitivity <20 ppm of analytes, selectivity at 50 ppm of terpenes, repeatability and reproducibility were thoroughly adjusted prior to online monitoring. Linearity in reversible responses over a wide concentration range <20–250 ppm has been achieved. Discrimination between molecules of similar molar masses, even for isomers, e.g. α-pinene and β-pinene is possible. The array has proven its sensitive and selective properties of sensor responses (20–1,200 Hz) for the difference of fresh and dried herbs. The sensor data attained was validated by GC-MS, to analyze the profiles of sensor emanation patterns. The shelf-life of herbs was monitored via emanation of organic volatiles during a few days. Such an array in association with data analysis tools can be utilized for characterizing complex mixtures.

## 1. Introduction

Terpenes are hydrocarbons released into environment from herbaceous vegetation. These biogenic volatile compounds, which include monoterpenes, sesquiterpenes and oxygenated terpenes [1], affect the global radiation balance due to the vigorous reactions of their oxidative products with ozone and OH in the troposphere [2]. On the other hand these terpenes are responsible for the characteristic whiff and fragrance in plants and are very important candidates from the sensing point of view. It is vital to investigate freshness, shelf-life and usability of these plants because many terpenes emanate from herbs used in fresh and dried form as spices and flavoring agents in food processing. This sensing is also essential for human health [3], as well as for atmospheric [4] and environmental [5] concerns. Human thresholds for odor, nasal pungency, nasal localization, and eye irritation to some of the selected terpenes, which enhance the need of highly sensitive and selective methods for quantification of these compounds, have been described by Cometto *et al.* [6]. Generally, these analytes are extracted, pre-concentrated, and analyzed by gas chromatography/mass spectrometry (GC-MS) [7], gas chromatography flame ionization detection (GC-FID) [8], accelerated solvent extraction (ASE) GC-MS [9], solid-phase microextraction (SPME) GC-MS, GC-FID [10] and automated thermal desorption (ATD) GC-MS [11]. However, these methods are quite expensive and require expert trained personnel. A favorable solution to this problem might be to identify some characteristic ingredients which will give a hint for the quality of the herbs. A simple analytical technique is desirable in order to provide on-line detection. Arrays of chemical sensors based on multichannel quartz crystal microbalances (QCM) *i.e.*, electronic noses [12,13] and tongues [14,15] are a quite suitable option for continuous and online monitoring in this scenario.

Sensor arrays can be designed considering two aspects: firstly, sensing materials need to be low cost, facile to synthesize, stable, and highly sensitive as well as selective. Secondly, transducer elements should be sensitive to minute fluctuations of analyte concentrations and being portable and rapid in response. To address first issue, artificial recognition materials generated via molecular imprinting [16] provide highly efficient applications in sensor architecture that mimic the capabilities of biological noses and can still overcome the inherent limitations of their natural counterparts, especially poor chemical and thermal stability. Molecularly imprinted polymers (MIPs), one of the most promising recognition materials for e-nose, have been applied since their first introduction [17] as highly selective coatings with excellent binding efficiency of analyte molecules. Polymers are synthesized in the presence of analytes acting as templates. High cross-linking will guarantee sufficient stiff cavities for selective analyte inclusion. With this approach, even isomers can be distinguished with sufficient selectivity. QCM represents a kind of transducer which exploits the benefits of MIPs without label-free detection [18]. Mass loading of QCM is visualized by frequency shift which can be monitored by an oscillator circuit. This kind of measurement shows a universal applicability, robustness, portability and high sensitivity. Usually, a wide linear range of sensor responses can be observed. Thus, MIP-QCM sensor arrays [18] can be used for a large variety of analytical problems of interest.

**Figure 1.**
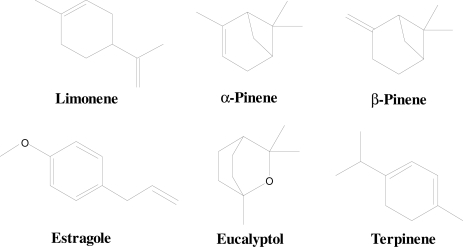
Structures of terpenes used for molecular imprinting and chemical sensing.

Some attempts [19,20] have already been made in order to detect terpenes in herbs using MIP-based QCM, and selectivity has been identified as the most important challenge in this field. During the course of this investigation we developed QCMs with six channels, coated with MIPs for continuous and online monitoring of terpenes emanated from fresh and dried species of the Lamiaceae family *i.e.*, rosemary (*Rosmarinus Officinalis L.*), basil (*Ocimum Basilicum*) and sage (*Salvia officinalis*). Additionally, headspace GC-MS analysis of the herbs was carried out. Both devices were calibrated with selected terpenes showing relevance in different aspects [21–24]. Our measurements will guarantee freshness. This quality control of fresh and dried herbs will reveal the shelf life of these herbs after several days of measurements. Polystyrene (PS) and divinylbenzene (DVB) copolymers were used for molecular imprinting by terpenes of interest *i.e.*, α-pinene, limonene, eucalyptol, β-pinene, terpinene and estragole. Figure 1 shows the chemical structures of all six templates which were selected on the basis of headspace GC-MS analysis of volatile ingredients of herbs before proceeding to record real time measurements. Optimization is performed in respect to layer composition concerning cross linkers as well as for layer heights to attain optimized results. Despite to close resemblance in structures of the emanated terpenes, MIPs will show high sensitivity and selectivity towards their corresponding templates due to geometrical adaption.

## 2. Results and Discussion

### 2.1. Layer Optimization

**Figure 2.**
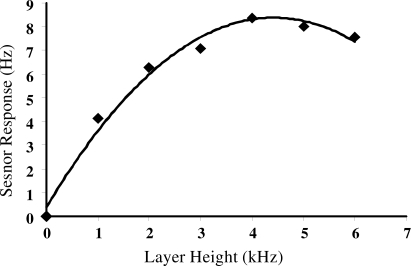
Variation of sensor response with changing layer heights of terpinene MIP at 50 ppm.

The relationship of MIP composition in relation to the sensor signals obtained is revealed in Table 1. A normalized response of the MIP-layers to limonene and eucalyptol respectively is given. Optimized signals were obtained with a 1/1.5 styrene/DVB copolymer composition, using 10 μL of templates, whereas other layers and template concentrations show lower responses. It is obvious that an increase in crosslinker will lead to a more rigid polymer capable of functionalizing the material in respect to cavities. Furthermore, the amount of template is very decisive. Only a few number of template molecules will generate less interaction centers in the coating, thus a diminished sensor effect will result. A high amount of imprinted molecules causes a dilution of the monomers and an effective polymerization is hindered. The sensor properties of the coatings are tailored according to these ideas [16]. We used different layer heights of polymers in the range of 1–6 kHz, where 1 kHz corresponds to a thickness of 40 nm. This variation of sensor signal is demonstrated in Figure 2 and an optimum layer height is observed. Obviously this finding can be attributed to a bulk behavior since the response increases in parallel to layer height. At a layer height of approximately 4–5 kHz a plateau formation is followed by some hint for a slight decrease in frequency response. This behavior is easily understood by analyte diffusion in the interior of the layer. Thin layers incorporate analytes easily due to the pores which are generated additionally by the templates. An increasing layer height will statistically block the excess to the diffusion channels and thus the behavior in Figure 2 is explained.

### 2.2. Selectivity Pattern of the E-nose

**Figure 3.**
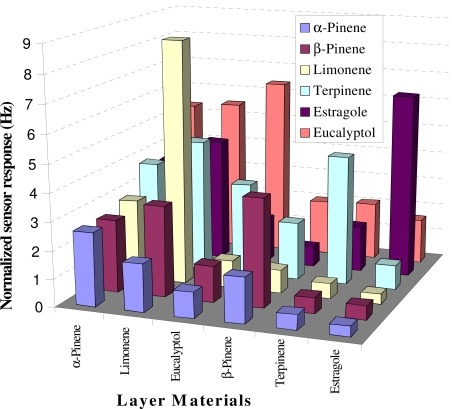
Selectivity profile of e-nose at 50 ppm.

Figure 3 shows the selectivity pattern of all polymer layers at 50 ppm concentration of each analyte. The sensor array is alternatively exposed to different analytes with the same concentration and normalized according to layer height. The astonishing observation is the capability of the sensor array to distinguish isomeric compounds as α-pinene and β-pinene. Similarly all other layers showed maximum selectivity towards their corresponding templates. Thus the designed sensor array proved to be sensitive and selective for online monitoring of terpenes emanated from fresh and dried herbs. In addition, a sensing ability of sensor for analytes with similar structures is beneficial in regard of analyzing complex mixtures. Such problems are best addressed by multivariate analysis and the collected data should be analyzed through modern data analysis tools which are important part of today’s electronic bio-mimetic instruments.

### 2.3. Sensitivity Profile Established from Fresh and Dried Herbs

**Figure 4.**
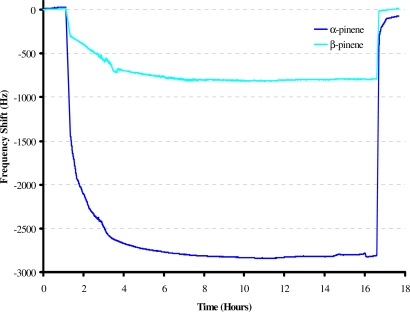
Emanation from fresh rosemary by MIP layers imprinted with α-pinene and β-pinene.

This sensitive and selective e-nose was used for online monitoring of fresh and dried rosemary, basil and sage. For fresh herbs each measurement was continued for 5–7 days, whereas for dried ones it took three days. The time of each measurement depended upon the abundance of terpenes recorded by headspace GC-MS analysis. Figure 4 shows the sensitivity profile of some original data on terpenes emanated from fresh rosemary; the “on-line” monitoring was performed via 10 MHz QCM. The responses of two sensors were chosen which were templated with β-pinene and α-pinene, respectively. It is observed that each sensor shows a characteristic response depending on the template molecule.

Imprinting e.g., with β-pinene yields frequency shifts by more than a factor of two compared to α-pinene. After a stable baseline was established a constant vapor pressure of rosemary emanations was reached, and the frequency shift is lowered by ≤2,800 Hz and ≤700 Hz for the layers. Thus, a stable sensor behavior was observed for several hours, leading to a highly reproducible sensor signal at the end point. Sometimes minute fluctuations of sensor responses were monitored, depending upon temperature changes which can be eliminated by differential measurements. A similar trend was observed for all fresh and dried herbs.

During online monitoring, small humidity effects of ≤40–60 Hz were observed, which are negligible in comparison to actual signals of fresh rosemary. Former investigations had revealed similar humidity cross sensitivities, when copolymers based on styrene/divinylbenzene were used [18]. Additional improvements resulted from a QCM coated with polyvinyl alcohol, which show strong hydrophilic properties. Thus, a nearly selective humidity sensor is available, which can be used for taking into account variations of ambient surroundings.

**Figure 5.**
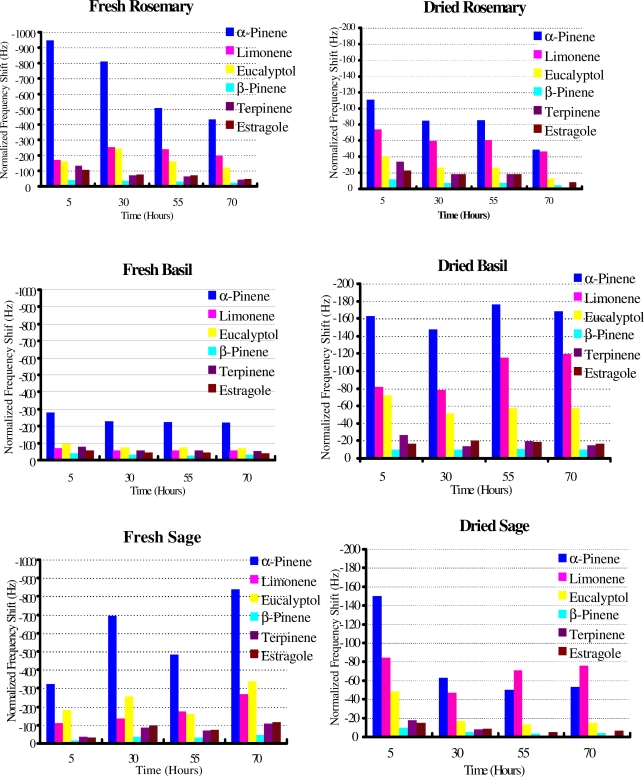
Terpene emanation patterns from fresh and dried herbs.

In Figure 5 emanation patterns of terpenes from all fresh and dried herbs considered as a function of time are shown. The data generated by sensor array measurements shows an obvious difference between terpenes emanated from fresh and dried herbs as high responses of α-pinene of 310–950 Hz and 110–176 Hz from fresh and dried herbs were observed respectively. The second major contribution of limonene and eucalyptol *i.e.*, 250–350 Hz from fresh and 70–120 Hz from dried herbs was observed.

A third major pattern was obtained from terpinene and estragole *i.e.*, 100–132 Hz from fresh and 23–33 Hz from dried herbs. The lowest amounts ≤44 Hz and ≤12 Hz of β-pinene have been observed from all the fresh and dried herbs respectively. These responses basically correspond to the amount of terpenes emanated from the herbs. As the measurement proceeded further these responses decreased towards lower values in the same fashion. The most innovative feature of the sensor array is the achievement of sensitivity and selectivity for real time and online monitoring of emanated terpenes under ambient conditions efficiently and quickly. Furthermore it is sensitive enough to distinguish between isomers of a compound like α-pinene and β-pinene. A linear behavior is observed between the sensor signals and the amounts of emanates and each layer is responding differently towards its analyte *i.e.*, greater the amount of analyte, higher the signal and *vice versa*. This behavior can be explained by the mass sensing nature of the transducer and structural properties of analytes. Four isomers of terpene family *i.e.*, α-pinene, β-pinene, limonene and terpinene have similar molar masses and formulas. There could be interferences due to these similarities but due to their structural dissimilarities like difference in spatial arrangements of double bond and functional groups, the stereochemistry of each member justify its interaction towards its respective MIP-coated e-nose layer. As for an example α-pinene differs from β-pinene not only by position of double bond, but also the steric hindrance of the methyl group of α-pinene does not allow it to interact with cavities generated by the β-pinene template. Similar behavior is predicted in case of limonene and terpinene.

**Figure 6.**
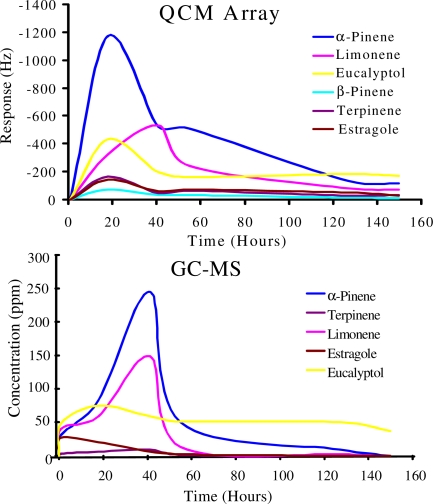
QCM array and GC-MS comparison of terpenes from fresh rosemary.

The data obtained from the e-nose was validated by headspace GC-MS analysis after regular intervals of time, which confirmed the terpene emanation patterns. Both devices were calibrated with selected terpenes. The data obtained from GC-MS also supports the information provided by the sensor array as in Figure 6.

A quite similar emanation behavior was observed from both devices when exposed to fresh and dried herbs. Vapors of terpenes emitted from fresh and dried herbs were transferred through a heated Teflon sample-tube for GC-MS analysis. The data obtained showed that a maximum amount ≤250 ppm of α-pinene, ≤150 ppm of limonene, and ≤75 ppm of eucalyptol were detected whereas ≤20–30 ppm of terpinene, and estragole were observed in fresh rosemary. Consequently the minimum amount of the different terpenes that can be detected by the QCM sensor array in its defined position with relation to the emanating herbs at ambient environmental conditions was estimated to lies beyond at least 20 ppm. From these observations, the working strategy of the e-nose efficiently enlightened its mass sensing capability and it can be concluded that the proposed e-nose is ideal and cost effective to work for online measurement.

### 2.4. Effect on Selectivity of E-nose at Maximum Response from Fresh and Dried Herbs

**Figure 7.**
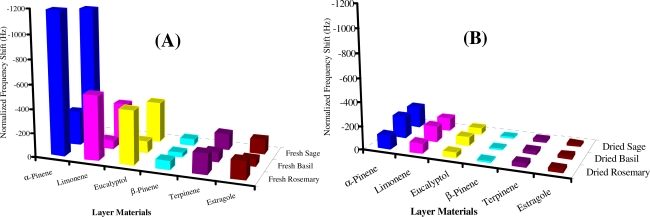
Selectivity patterns of e-nose, (A) Emanated terpenes from fresh herbs, (B) Emanated terpenes from dried herbs. Normalized at layer height = 40 nm, actual layer height = 180–240 nm.

Figures 7(A and B) show the maximum sensor responses observed from fresh and dried herbs. Higher sensor signals were recorded from fresh herbs as compared to dried. In the case of fresh herbs, rosemary and sage showed the maximum responses *i.e.*, 1,183 Hz and 1,092 Hz for α-pinene, 533 Hz and 292 Hz for limonene, 445 Hz and 338 Hz for eucalyptol, 74 Hz and 51 Hz for β-pinene, 170 Hz and 132 Hz for terpinene, 149 Hz and 117 Hz for estragole respectively. The maximum signals recorded from fresh basil were, 281 Hz for α-pinene, 68 Hz for limonene, 98 Hz for eucalyptol, 38 Hz for β-pinene, 77 Hz for terpinene and 55 Hz for estragole, which are lower than for rosemary and sage. This distribution of maximum sensor signals for terpenes represented selective attitude of the MIP-QCM based e-nose. Interestingly dried basil showed higher sensor signals for α-pinene (183 Hz), limonene (130 Hz) and eucalyptol (74 Hz), whereas dried rosemary showed maximum amounts of terpinene (36 Hz) and estragole (24 Hz) in terms of sensor signals. Comparable amounts of β-pinene (16–24 Hz) were observed from all dried herbs. Dried sage on the other hand gave higher sensor signals *i.e.,* 170 Hz for α-pinene, 108 Hz for limonene and 54 Hz for eucalyptol as compared to dried rosemary *i.e.,* 111 Hz for α-pinene, 81 Hz for limonene and 44 Hz for eucalyptol. These patterns reveal again the selectivity behavior of the QCM based e-nose.

### 2.5. Determination of Shelf-Life and Usability of Fresh and Dried Herbs via E-Nose

Freshness evaluation of a substance is of great interest in food industry. E-noses of various sensing materials have already been used in this regard [25]. Substantial achievements were also obtained in this field by using MIP coated QCM based e-nose for determination of volatile constituents compared to the classical, bulky and expensive chromatographic methods [26]. Aroma contents of herbaceous plants can be used to assess their freshness and shelf-life. The sensor signals recorded as a function of time from the emanated terpenes is the powerful tool to determine the shelf-life of fresh and dried herbs. In fresh rosemary and sage maximum values were achieved earlier, which indicated high amounts of terpenes, especially α-pinene, limonene, estragole *etc.*

**Figure 8.**
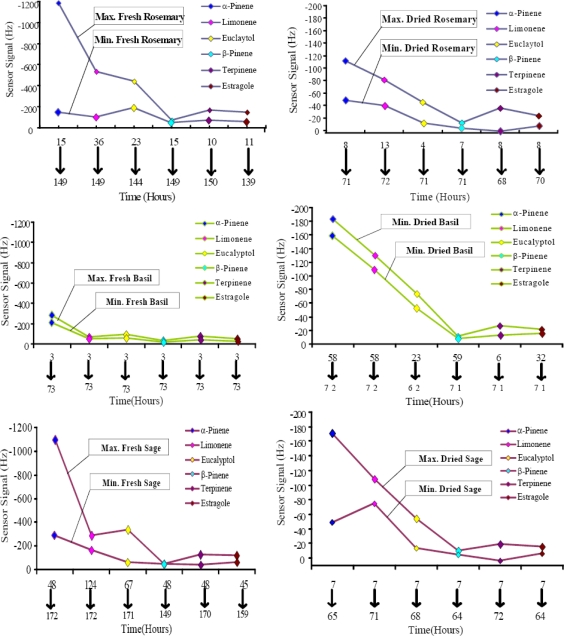
Comparison of maximum and minimum responses.

Figure 8 shows a comparison for fresh and dried species of herbs from maximum to minimum sensor signal. In case of fresh rosemary high sensor responses for selected terpenes *i.e.*, 1183 Hz for α-pinene, 533 Hz for limonene, 445 Hz for eucalyptol, 74 Hz for β-pinene, 170 Hz for terpinene and 149 Hz for estragole were observed in between 40 hours, whereas the minimal sensor signals (9–157 Hz) were observed till the 150th hour. This suggests that the best time of its use under usual environmental conditions is within first 36 hours after being removed from the garden.

While considering the behavior of dried rosemary, maximum emanation (12–111 Hz) occurred in first 15 hours and lead to lower sensor signals (04–48 Hz) till 72 h. Its higher signal values are equivalent to lower values of fresh rosemary indicating the dryness of herb due to emanation of volatile constituents. Dried rosemary can be used for longer time as microbial activity will be lower due to negligible amount of moisture. A similar kind of trend is observed from fresh and dried sage. Fresh basil in contrast showed its maximum sensor signals (37–281 Hz) for all terpenes after three hours of measurement and then lead to similar sensor signals, especially for α-pinene (219 Hz) and limonene (56 Hz), till the end of measurement. These identical sensor signals from high to low *i.e.*, (11–183 Hz) and (10–161 Hz) for all emanated terpenes were also observed in dried basil, including α-pinene and limonene. Focusing on shelf life, fresh herbs have shorter life compared to dry herbs. This study encompasses the efficacy of the designed e-nose in developing artificial receptors for real life applications.

## 3. Experimental Section

### 3.1. Materials and Chemicals

Quartz crystal sheets (f = 10 MHz) were purchased from Zheijiang (China) and the gold paste for screen printing of electrode structure was taken from Heraeus, (Germany). Limonene (98%-Fluka), α-pinene (98%-Aldrich), eucalyptol (98%-Fluka), β-pinene (80%-Fluka), α-terpinene (85%-Aldrich), estragole (99%-RDH), and diphenyl methane (99%-Aldrich) were used as received, styrene (99.5%-Fluka) and divinyl benzene (DVB-98%-Fluka) were extracted with 0.1 M NaOH to remove stabilizer, while azobisisobutyronitrile (AIBN-98%-Aldrich) was re-crystallized from methanol before use. Fresh rosemary, basil and sage were taken from a house garden whereas dried herbs were purchased from local supplier.

### 3.2. Measuring Setup

**Figure 9.**
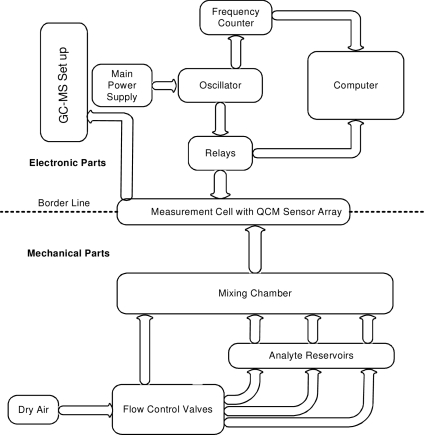
Flow sheet diagram of measuring components of the QCM based e-nose.

The main components involved in the basic measurement setup are described in Figure 9. For frequency measurements, a network analyzer (E5062A from Agilent Technologies) and a frequency counter (5385A from HP) were used. Sensor signals were recorded using self made oscillator circuit provided with a switch of relays to convert different array electrodes to the oscillator circuit. The chemical sensor array chamber is connected to air and analyte inlets in which, flow of air through various analyte reservoirs was controlled via mass flow controllers (Tylan-RO7020) in order to allow calculated amounts so as to generate required concentrations. Whole equipment, excluding electronic parts, was thermostated by circulation of water at specific temperature as required. Although, a source of dried and compressed air was used throughout the experiments, yet it is impossible to fully get rid of inherent humidity which was 0.5–2%RH, when measured by hygroscope (Rotronic DV-2).

### 3.3. Head Space Gas Chromatography-Mass Spectrometry (HS, GC-MS)

The data obtained from the MIP coated QCM sensor array or e-nose was validated by headspace GC-MS analysis after regular intervals of time in every measurement of fresh and dried herbs. An Agilent GC-MS instrument consisting of a 6830 N gas chromatograph, 5973 N mass spectrometric detector and an auto sampler from COMBI PAL was used. Sampling of air containing vapors of emanated terpenes was carried out using heated Teflon coated sample line. A capillary column (J&W cross-linked 5% phenyl methyl siloxane −30 m length in a diameter of 0.25 mm, 0.25 μm coating) was used for separation of sample components. Each analysis included a temperature programme starting from 100 °C for 4 min followed by a ramp of 5 °C/min and reaching to a plateau of 250 °C in splitless mode with He as carrier. The organic vapors are quantitatively and qualitative identified by total ion current signals using the built in software’s library.

### 3.4. Manufacturing of Sensor Array

**Figure 10.**
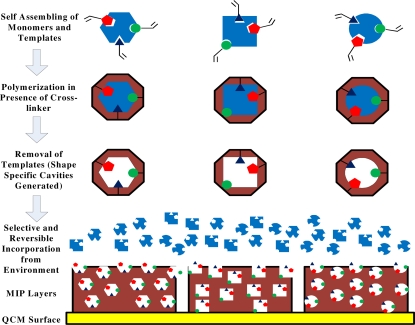
Generation of selectivity through molecular imprinting.

The sensor array consisted of two quartz sheets; each containing three gold electrodes and electrode structure was printed on the quartz surface by screen printing procedure. MIPs were synthesized by mixing 30 μL styrene as functional monomer, 70 μL DVB as cross-linker, 2 mg AIBN radical initiator and 5 μL diphenylmethane as porogen. Three hundred μL of template was added to this mixture for imprinting as optimized from a number of observations, so six MIPs were prepared with different terpenes. Finally, each mixture was polymerized for 40 min at 70 °C. Layers of these polymers were coated on electrode by spin coating. To avoid mixing of the polymers, a specially designed polydimethylsiloxane (PDMS) block consist of two pieces was used. The upper piece contained three holes which just fit over three electrodes of the QCM. Quartz plate is fixed in between these two pieces and the required quantity of polymer solution is poured into the holes before spinning it with a specific speed. For polystyrene layers, 5 μL of each MIP solution was used and spinned at 2,000 r/min for 45 s. The resulting layers leads to a frequency shift of 1–6 kHz which corresponds to a layer height of 40–240 nm. Layers were dried overnight at room temperature to evaporate the template leaving behind specifically adapted cavities. A graphical representation of the imprinting procedure is given in Figure 10.

### 3.5. Online Measurement Strategy

For evaluation of the sensor responses, the sensor array was first exposed to dry air to achieve a stable baseline, then it was exposed to known analyte concentration of each selected terpenes ranging from 10–100 ppm for 15–30 min until a stable response was obtained at maximum re-inclusion. For regeneration of array it was again exposed to dry air. In parallel headspace GC-MS analysis was performed from the chamber containing sensor array. Similarly the online monitoring of terpenes emanation from fresh and dried herbs was carried out. During this measurement headspace GC-MS analysis was also performed time to time which gave information about emanates. When both devices represents negligible amounts of terpenes then sensor array was exposed to dry air again to get stable reversible base line. The data obtained from selected terpenes and terpenes emanated from fresh and dried herbs were used to establish a correlation between GC-MS and sensor responses recorded. Quantitative information about emanated terpenes from fresh and dried herbs was determined by creating an artificial neural network (ANN) with the MatLab software by MathWorks [18]. For the neural network (NN) a routine script was used to recognize analyte concentration patterns within frequency signals. Thus the dimensions and algorithms of the NN had to be designed to process the sensor data generated over time and deliver concentration values within a predefined standard deviation. This was set to the predicted values that could be compared to GC-MS data. The network was designed with 33 nodes in the second layer after calculations for optimizing predicted correctness and processing speed which depends upon the matrix dimensions. For training the network calibration data was used, which was achieved by subjecting our sensor arrays to clearly defined concentrations of analytes/air mixtures for giving related data signals. As the NN should produce concentrations after processing the signals we trained it by using the signal-matrices for the input into the NN and comparing the concentration-matrices as the output during training where we allowed the system to run for as much as 300 to 600 epochs. In this way the correctness was already trimmed for validation purpose to be in the right sequence as well as for the final simulation. The learning algorithms used were all back-propagation oriented and do react over proportionally to reach the steepest descend.

## 4. Conclusions

A 10 MHz QCM based e-nose containing polystyrene MIP layers has been successfully applied to generate sensitivity and selectivity for terpenes released from fresh and dried species of Lamiaceae family *i.e.*, rosemary (*Rosmarinus Officinalis L.*), basil (*Ocimum Basilicum*) and sage (*Salvia officinalis*). For this purpose, a unified approach of synthesis process is adapted to optimize the composition of polymer. Layer heights (40–240 nm) were optimized, which showed selectivity at 50 ppm of the selected terpenes. A wide range of sensor signals from <20–1200 Hz were obtained from terpenes emanated from fresh and dried herbs. Sensor responses recorded for isomers *i.e.*, α-pinene (111–1183 Hz) and β-pinene (10–74 Hz) emanated from fresh and dried herbs also emphasized the selective behavior of e-nose. Similarly sensor responses of limonene, eucalyptol, terpinene and estragole attained from fresh and dried herbs also revealed sensitivity and selectivity. The sensor data obtained from the e-nose was validated by GC-MS which reveals that the designed e-nose is sensitive enough to detect <20 ppm of emanated terpenes. Fresh herbs showed 6–10 time higher sensor signals for emanated terpenes than dried herbs. MIP-QCM sensors possess high potential for solving problems of analyzing resembling constituents of a complex mixture. Moreover, this miniaturized multichannel QCM based e-nose elegantly behaved as an artificial receptor which can be best compared to human olfactory systems. Furthermore, a remarkable sensitivity and selectivity is achieved while comparing fresh and dried herbs, even at the isomeric level e.g., α-pinene and β-pinene. As emanation pattern of terpenes obtained from herbs is the function of time so, it can be useful to determine the freshness, usability and shelf-life of herbs. Simplicity and efficacy of this system suggests that arrays should be used for real time analysis of complex mixtures e.g., composting exhaust, herbaceous odorant emanation, or air quality as a whole. For this purpose, sensors will have to be calibrated by selecting marker compounds of major significance and data analysis systems will have to be installed for real time evaluation. Electronic noses, thus manufactured, can be used for a large number of industrial applications.

## Figures and Tables

**Table 1.** Sensor responses to limonene and eucalyptol as function of MIP composition.

**Layers**	**Composition (μL)**			**Normalized Response (Hz)**		

	**Styrene**	**DVB**	**Template**	**25 ppm Limonene**	**50 ppm Eucalyptol**	

1	1	1.5	0	0.98		1.23
2	1	1.5	5	3.81		4.84
3	2	1.5	5	3.13		4.17
4	1	1.5	10	6.92		6.39
5	2	1.5	10	4.81		3.45
6	1	1.5	15	5.41		6.01
7	2	1.5	15	4.97		5.77
